# Tactile Augmentation of Material Classification via Imperceptible On‐Skin Triboelectricity Collection

**DOI:** 10.1002/advs.202500217

**Published:** 2025-07-02

**Authors:** Junting Huang, Stanley Gong Sheng Ka, Haydn Cheong, Yaru Zhang, Daping Chu, Sohini Kar‐Narayan, Wenyu Wang, Yan Yan Shery Huang

**Affiliations:** ^1^ The Nanoscience Centre University of Cambridge 11 JJ Thomson Avenue Cambridge CB3 0FF UK; ^2^ Department of Materials Science and Metallurgy University of Cambridge Cambridge CB3 0FS UK; ^3^ Department of Engineering University of Cambridge Trumpington Street Cambridge CB2 1PZ UK; ^4^ Centre for Photonic Devices and Sensors Department of Engineering University of Cambridge Cambridge CB3 0FA UK; ^5^ Department of Institute for Materials Discovery University College London London WC1E 6BT UK; ^6^ Thrust of Smart Manufacturing Hong Kong University of Science and Technology (Guangzhou) Guangzhou 511458 China

**Keywords:** fibers, material differentiation, tactile augmentation, triboelectricity

## Abstract

Harnessing intrinsic triboelectric signals from human skin holds promise for enhancing tactile perception. However, collecting these signals without disrupting normal skin functions and convoluting motion artifacts remains challenging. Additionally, person‐to‐person signal variance complicates data processing. In this study, it is demonstrated that triboelectric signals generated from touch can be imperceptibly collected and processed using a machine learning model to achieve tactile augmentation. When one hand contacts and rubs against a target object, charge transfer occurs between the skin and the object's surface. By placing a substrate‐less microfiber electrode on the finger of the other hand, a body‐coupled triboelectric circuit is formed to collect these signals, which contain material‐specific features such as amplitude and peak ratio. A machine learning technique is developed to process the triboelectric signals, enabling the classification of six different materials with a prediction accuracy of ≈95%. The material differentiation model is further validated across different users, achieving an overall accuracy of ≈88 %, illustrating the potential of utilizing the body‐coupled triboelectric circuit for tactile augmentation.

## Graphical abstract

Human skin inherently forms a body‐coupled triboelectric circuit that enables the generation of triboelectric charges through contact between the skin surface and various materials. Integrated with an on‐finger, substrate‐free microfiber electrode, the material‐specific and personalized triboelectric signals can be imperceptibly collected for machine learning‐based material identification, advancing the paradigm for human tactile augmentation.

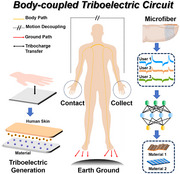

## 1 Introduction

Human finger skins possess an inherent tactile perception that can infer the material properties and surface textures upon touch.^[^ ^1^, ^2^ ^]^ The ability to harness and transduce such tactile perception from the skin into electronic signals could facilitate future advancements in wearable devices^[^ ^3^, ^4^ ^]^ and human‐machine interactions.^[^ ^5^, ^6^ ^]^ However, the proficiency of inherent skin tactile perception in differentiating the material generics and surface textures might be limited when other perceptions, such as proprioception, visual supports, and thermal cues, are absent.^[^ ^7^, ^8^ ^]^ Thus, further augmenting the skin's tactile perception with enhanced material differentiation ability could benefit applications such as assisted rehabilitation training or disability assistance.^[^ ^9^, ^10^ ^]^ Ideally, the transduction and augmentation of human finger skins’ tactile perception should be operated imperceptibly to maximize comfort and preserve intrinsic physiological functions.

Electronic skins (e‐skins) with triboelectric sensing functions could enable material and surface texture differentiation through friction‐induced charge transfer.^[^ ^11^, ^12^, ^13^, ^14^, ^15^, ^16^, ^17^ ^]^ The triboelectric signals generated during the interactions necessitate advanced data processing tools, particularly machine learning techniques, for effective feature extraction.^[^ ^18^, ^19^ ^]^ For instance, machine learning techniques can establish temporal sequences and channel correlations in triboelectric signals, deciphering characteristics involving roughness, geometry, pressure magnitude, thermal properties, and related parameters for comprehensive interpretation of tactile stimuli.^[^ ^20^, ^21^ ^]^ By taking advantage of the machine‐learning‐driven analysis, several triboelectric e‐skin systems have demonstrated biomimetic multi‐modal tactile sensing with precise detection of pressure and vibration, gesture recognition, geometry identification, and enhanced object classification, exhibiting significant potential of tactile augmentation.^[^ ^22^, ^23^, ^24^ ^]^ Therefore, integrating triboelectric e‐skins with machine learning represents a low‐cost, self‐powered solution toward tactile sensing,^[^ ^25^, ^26^, ^27^ ^]^ which promises applications to collaborative robots and humanoids with biomimetic and enhanced environmental perceptions.^[^ ^28^, ^29^, ^30^, ^31^, ^32^ ^]^ However, these triboelectric e‐skins may not be the most suitable when applied onto human finger skins for tactile perception enhancement, since they are normally composed of solid film‐based structures,^[^ ^28^, ^33^, ^34^, ^35^ ^]^ which would cover skin surfaces and inhibit normal physiological functions such as perspiration and sensations. In addition, constant friction and scratching of the e‐skins could inevitably lead to device degradation, resulting in performance deterioration or failure over long periods of use.^[^ ^36^, ^37^, ^38^ ^]^

Instead of relying on external device layers for human tactile augmentation, harnessing the intrinsic triboelectric signals from human finger skin upon surface friction and contact could potentially offer an efficient and comfortable route for human tactile augmentation. The human skin epidermis intrinsically serves as a self‐regenerative dielectric layer for durable triboelectrification, while the underlying dermis functions as an ionically conductive layer connected throughout the entire human body.^[^ ^39^, ^40^ ^]^ The skins could naturally serve as a body‐coupled triboelectric nanogenerator, enabling electrical signal emanation via surface friction with another object.^[^ ^41^, ^42^ ^]^ The intrinsic triboelectric signals generated from human skin could vary between individuals because of the physiological discrepancies in human skin, such as hydration level, elasticity, roughness, and composition.^[^ ^43^, ^44^ ^]^ Thus, it could be important to establish a user‐specific triboelectric signal library for body‐coupled tactile perception transduction and augmentation.^[^ ^45^, ^46^ ^]^ Overall, harnessing the intrinsic body‐coupled triboelectric signals from human skin could potentially inspire sustainable and imperceptible tactile augmentation. However, challenges remain in the lack of an effective and minimally invasive method for collecting the body‐coupled triboelectric signals while enabling signal analysis for tactile augmentation.

Here, we show that the body‐coupled triboelectric signals from human finger skins could be collected with imperceptible microfiber electrodes, and the user‐specific signals were processed with a machine learning model to establish user‐specific tribo‐characteristics for material recognition. Using human skin as the triboactive layer, the body‐coupled triboelectricity was generated when the skin was in contact and produced friction with the targeted object surfaces. To collect these signals, a microfiber electrode was printed on the other hand across the human body using a previously reported method.^[^ ^47^ ^]^ In this design, the signal‐collecting electrode, composed of substrate‐less microfibers of an open network, was physically decoupled from the frictional and vibrational movements. Such a configuration could minimally impede the intrinsic physiological functions of the skin while maximally decoupling the motion artefacts. The body‐coupled triboelectric circuit was tested on six different materials, capturing signals with several user‐specific signal features. A machine learning model was developed to process the signals of six different materials for establishing user‐based tribo‐characteristics for tactile augmentation with up to ≈95% accuracy for material recognition. Finally, the established model was validated experimentally across all users for real‐time classification with an accuracy of ≈88%.

## 2 Results and Discussion

### 2.1 Body‐Coupled Triboelectric Circuit

**Figure 1:**
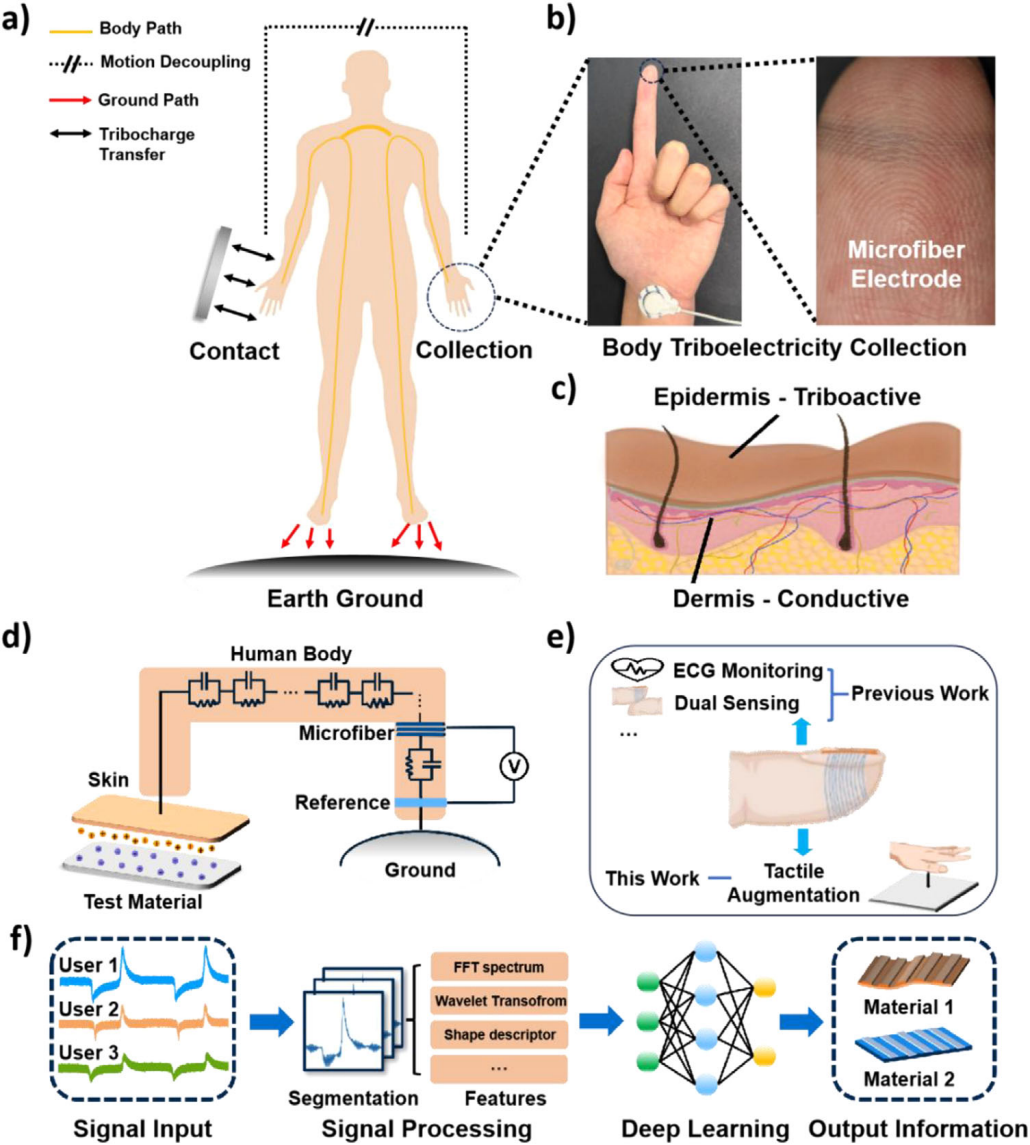
Microfiber electrode for the body‐coupled triboelectric circuit: a) Schematic illustration of the body‐coupled triboelectric circuit.^[^ ^43^, ^52^ ^]^ b) Photographic images of the working electrode of microfiber and the reference electrode of commercial gel, as well as a zoomed‐in picture of microfiber printed on the index fingertip. c) Skin configuration for dielectric epidermis and conductive dermis. d) The equivalent model of single‐electrode mode TENG for the body‐coupled triboelectric circuit. e) The multifunctional and imperceptible microfiber electrode for living system augmentation, including ECG monitoring, dual‐sensing, etc, in the previous work,^[^ ^47^ ^]^ and body triboelectric collection for tactile augmentation in this work. f) Triboelectric signals perceived by on‐skin microfiber electrodes for user‐specific material information capture and tactile augmentation through signal processing and machine learning techniques.

Inspired by the configuration of single‐electrode mode triboelectric nanogenerators (TENG),^[^ ^48^, ^49^, ^50^ ^]^ a body‐coupled TENG, was introduced using human epidermal skin as the triboactive layer. As depicted in **Figure** **1**a, the triboelectric agitation that occurred in the left hand, undergoing body path and ground path, was finally transmitted and collected by the right hand printed with the microfiber electrode. Detailed images of the microfiber electrode configuration are shown in Figure 1b, coupled with a reference gel electrode for body triboelectricity collection. The body's triboelectricity stems from the dielectric epidermis of human skin, which acts as a durable triboactive layer. The epidermis facilitates triboelectric charge generation upon contacting external objects. The dermal and subcutaneous layers beneath the epidermis act as ionically conductive layers for triboelectricity transmission.^[^ ^51^ ^]^ The triboelectric charges would then transmit across the human body (Figure 1c).

The entire system can be viewed as a single‐electrode mode TENG with an equivalent circuitry shown in Figure 1d. Upon contact with a target material, the triboelectric charge transfer between the skins and the target material would occur due to mechanical agitation. This process could occur in the reverse direction when the material was separated from the skins, resulting in a unique two‐peak triboelectric signal. The microfiber working electrode, printed on the finger of the other hand was used to record the triboelectric signals. It was shown that the position of the working electrode and reference electrode on different skin locations would negligibly affect the quality of triboelectric signal collection (Figure S1, Supporting Information). Therefore, by placing the microfiber working electrode on the other hand would physically decouple the mechanical agitation from the signal collection, potentially contributing to minimizing the motion artefacts and the wearing of the electrode. With the microfiber electrode printed on the fingertip, it could potentially function as a multifunctional sensing platform, integrating the triboelectric signal collection with the previously reported electrophysiology monitoring for tactile augmentation.^[^ ^47^ ^]^ (Figure 1e and Note S1, Supporting Information). With such body‐coupled circuit design, the distinct triboelectric responses of different users upon contact with different materials and surface textures could be measured. These signals, exhibiting user‐specific features, could then be analyzed by machine learning techniques for establishing user‐specific tribo‐characteristics, which transduce and augment human tactile perceptions (Figure 1f).

### 2.2 Microfiber Electrode for On‐Skin Triboelectricity Collection

**Figure 2:**
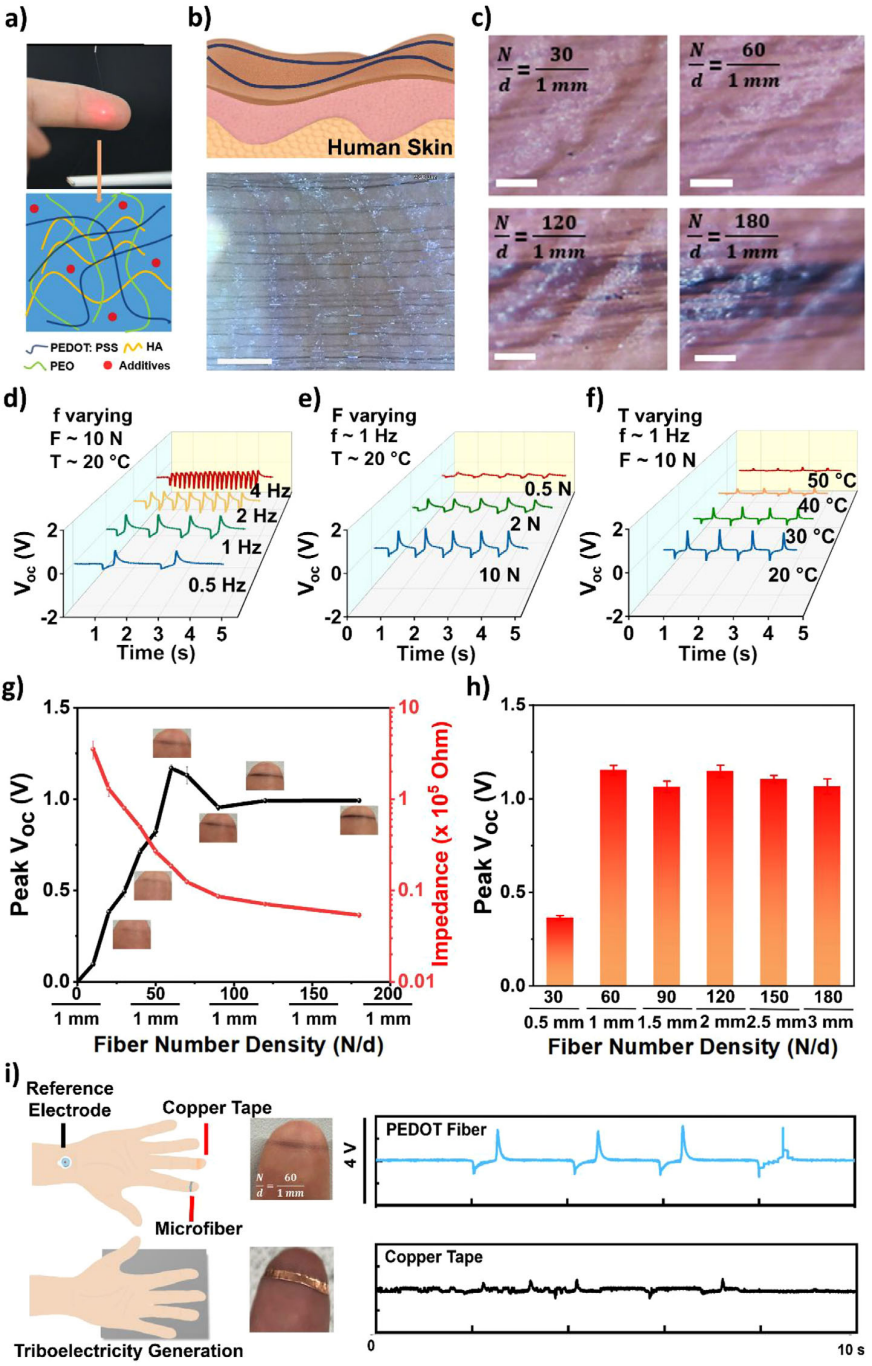
Fundamental characterization and optimization of microfiber electrode for body triboelectricity measurement: a) Photographic images of index fingertip tethered with microfiber solution through an orbital spinning cantilever and the main composition of the microfiber solution. (scale bar: 1 cm) b) Schematic illustration of microfiber for skin curvature adaptive deposition and high‐resolution optical microscopy images of on‐skin microfiber printed on skin. (scale bar: 520 µm) c) Photographic images of microfiber with different spined microfiber numbers over a constant width of 1 mm, with a scale bar of 0.2 mm d) V_oc_ under different frequencies, test material: Ecoflex. e) V_oc_ under different applied forces, test material: Ecoflex. f) V_oc_ under different temperature conditions, test material: PTFE. g) Peak V_oc_ and microfiber impedance measurement at 1k Hz over different microfiber numbers across a constant width of 1 mm. Error bars were calculated through standard deviation with at least 5 samples tested. Testing conditions: applying force:10 ± 2 N, frequency: 0.5 Hz, RT, RH. h) Peak V_oc_ with different spined microfiber numbers in controlling N/d as a constant of 60 mm^−1^. Error bars were calculated through standard deviation with at least 5 samples tested. i) Schematic illustration and results of comparison on microfiber electrode and copper tape for monitoring the body triboelectric signals.

The electrode for body triboelectricity collection was composed of substrate‐less microfiber arrays. The material and fabrication of the microfiber electrodes were reported in previous work.^[^ ^47^ ^]^ In brief, a biocompatible conducting polymer poly(3,4‐ethylenedioxythiophene) polystyrene sulfonate (PEDOT: PSS) was mixed with polyethylene oxide (PEO) and hyaluronic acid (HA). This combination ensured efficient fiber formation of PEDOT: PSS as the main functional phase. With the orbital spinning technique, the microfiber electrode was in situ deposited onto the fingertip adaptively (**Figure** **2**a). By tailoring the fiber parameters (N/d for fiber number/ fiber array width), the electrical and physical properties of the microfiber electrode could be controlled.^[^ ^47^ ^]^

Figure 2b shows a microscopic image of an array of microfibers deposited on the fingertip skin, revealing conformal skin adhesion. Figure 2c shows the pictures of the microfiber electrode on a fingertip with varying fiber number (N) over constant fiber array width (d) as fiber number density (N/d) increased from 0/1 mm to ≈180/1 mm. In general, N/d ≈60/1 mm would result in uniform fiber array distribution. The openness and substrate‐less feature of the microfiber electrode made it highly imperceptible, as exemplified by the fact that the fingertip with the microfiber electrode could still unlock the fingerprint password of a phone (Figure S2 and Video S1, Supporting Information).

We show that the triboelectric signal is collected from the body‐coupled circuit with a microfiber electrode by repeatedly patting the material with the hand in contact‐separation mode. As an open circuit, the triboelectricity signal is measured in open‐circuit voltage as V_oc_. Subsequently, the effect of hand patting frequency (f), applied force (F), and temperature (T) on the triboelectric signal collection was examined (Figure 2d–f). It was investigated that patting at ≈0.5 – 1 Hz produced clear and distinct wave patterns, and the increase in frequency resulted in signal overlap and diminished triboelectric output. It was observed that the applied force was generally proportional to the voltage output, and this is because greater mechanical agitation could lead to higher triboelectric charge transfer.^[^ ^53^, ^54^ ^]^ Besides, it was observed that temperatures above ≈40 °C would lead to a reduction of the signal amplitude, possibly due to the triboelectric charge dissipation by thermionic emission.^[^ ^55^ ^]^

The relationship between the triboelectric signal amplitude and the fiber number density (N/d) of the microfiber electrode was further investigated. It was found that N/d ≈60/1 mm would result in optimal signal collection. First, the N/d ratio to ≈60/1 mm could enable the skin contact impedance to achieve ≈10 kΩ at 1k Hz (Figure 2g), allowing for efficient charge transfer between the skin and the fiber electrode. Second, increasing the N/d ratio beyond ≈60/1 mm does not significantly contribute to enhancing the triboelectric signal amplitude (Figure 2g,h). This could be because increasing the fiber would lower the external load resistance (Figure S3, Supporting Information), thus diminishing triboelectric efficiency and voltage output.^[^ ^56^ ^]^ Thirdly, such density ensures uniform distribution without exhibiting significant fiber overlapping.

Further, a strip of copper tape was used as a comparison with the microfiber electrode for on‐skin triboelectricity collection. The triboelectric signals were simultaneously measured from the copper tape that adhered to the middle finger while the microfiber electrode (N/d ≈60/1 mm) was printed on the index finger. As shown in Figure 2i, the signals collected from the copper tape exhibited higher noise with a more unstable baseline and produced a lower voltage output due to its high skin contact impedance and sensitivity to mechanical and electrical noise (with gel electrode and microfiber electrode impedance compared in Figure S4, Supporting Information). In contrast, the triboelectric signals from the microfiber electrodes were more stable with lower background noise. This can be attributed to the intimate and stable mechanical interfacing between human skin and microfiber with lowered contact impedance, leading to enhanced triboelectric output.

### 2.3 Repairable Microfiber Electrode

**Figure 3:**
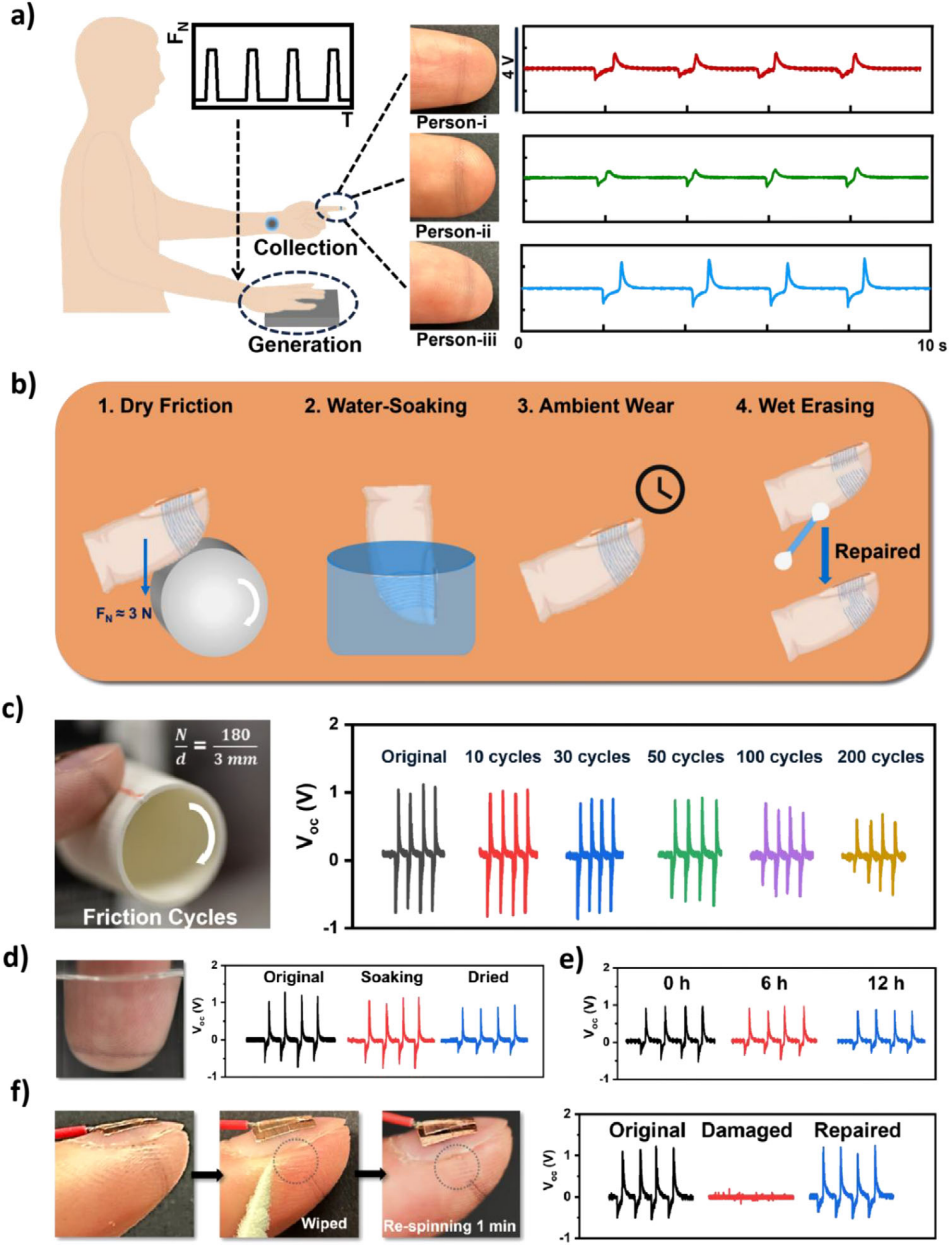
Adaptability, repairability, and stability of the microfiber electrode: a) Flexible microfiber for fingertip shape adaptation: illustration in microfiber tethering and signal generation at constant patting force and frequency. Photographic images of microfiber on the index fingertip of person‐i, person‐ii, and person‐iii with their personal voltage output via tapping the Ecoflex film. b) Schematic illustration of various environmental disturbances on the microfiber electrode. c) Wear resistance test of the microfiber electrode with an applied normal force of 2N and rotor linear velocity of 4 cm/s. V_oc_ output for microfiber electrodes under different wearing cycles (N/d ≈180/3 mm). d) Water resistance test by soaking the finger tethered with microfiber in deionized water and then drying in the air. V_oc_ for microfiber electrodes under wetting conditions (N/d ≈60/1 mm). e) V_oc_ output for microfiber ambient wear test in 0 h, 6 h, and 12 h (N/d ≈60/1 mm). f) Photographic images of the original microfiber electrode, the microfiber electrode erased by a wet cotton swab, and the repaired microfiber electrode. V_oc_ output for original, erased, and repaired microfiber (N/d ≈60/1 mm).

The stability and adaptability of the microfiber electrodes are important for personal triboelectric signal collection. **Figure** **3**a shows the triboelectric signal collection with microfiber electrodes (N/d ≈60/1 mm) printed on the index fingertips of different people. The microfiber electrodes, with conformal adhesion to curvilinear skins of different stiffness and roughness, are capable of collecting user‐specific triboelectric signals.

Although the microfiber electrodes were printed on the other hand and were not in direct contact with other objects, we still demonstrated stability against various environmental perturbations to mimic the daily wearing scenarios. The stability was tested via periodic dry friction, water‐soaking, ambient wearing, and wet erasing to simulate the conditions that fingertips may encounter daily, as shown in Figure 3b. The periodic dry friction experiment was conducted by pressing the microfiber‐tethered fingertip against a rotating plastic motor as photographed in Figure 3c and Video S2 (Supporting Information). The electrode exhibited no obvious macroscopic wear or significant signal reduction after 50 cycles (Figure S5, Supporting Information). After 200 cycles, it retained ≈62% of its original triboelectric output. As shown in Figure S6 (Supporting Information), under repetitive tapping up to 500 times with a silicone film, the microfiber electrode also illustrated no obvious degradation in collecting on‐skin triboelectricity. Figure 3d and Video S3 (Supporting Information) also proved that the on‐skin triboelectricity collection could be performed while the microfiber electrode was completely immersed in water. This could be attributed by the conformal fiber‐skin adhesion and the chemical stability of PEDOT:PSS in wet environments. A minor drop of ≈24 % of triboelectric output was observed after air‐drying, likely due to the dissolution of non‐cross‐linked additives.^[^ ^57^ ^]^ Ambient stability was confirmed through 12‐h ambient wearing (Figure 3e).

Although wet erasing or wiping could damage the microfibers for triboelectric collection, we show that the microfiber electrode could be on‐demand repaired through in situ microfiber re‐spinning (Figure 3f). The electrode's repairability was demonstrated by selectively erasing microfibers on the skin‐nail interface with a wet cotton swab. Re‐spinning the wiped area with microfiber solution for 1 min restored performance to ≈99% of the original output, highlighting the electrode's adaptability to environmental changes and repairability.

### 2.4 Material Differentiation by Body‐Coupled Triboelectric Signals Capturing

**Figure 4:**
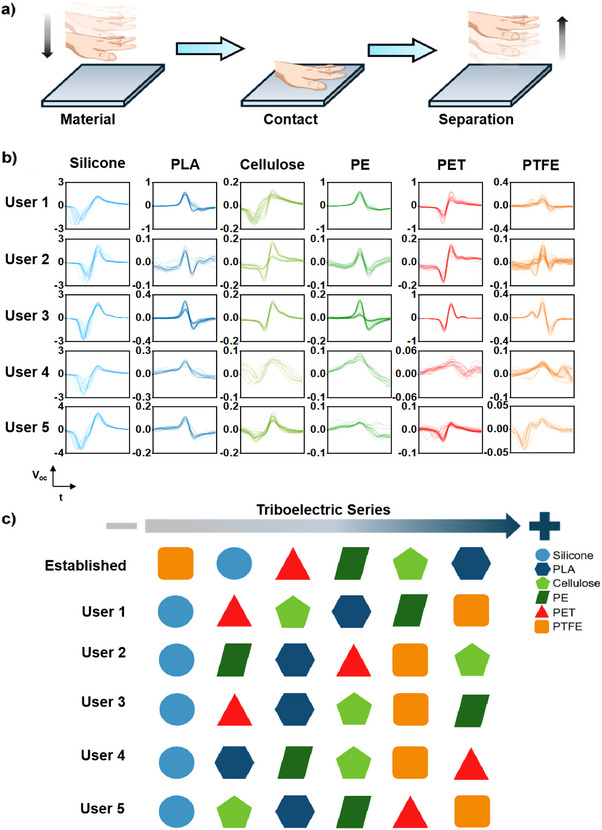
Microfiber electrodes for materials information capture: a) Schematic illustration of the body‐coupled triboelectric generation via contact‐separation mode by tapping the material on and off. b) The open‐circuit voltage output of all materials (silicone, PLA, cellulose, PE, PET, and PTFE) measured by all users in the condition of an applied force of ≈0.3 N, f ≈1 Hz, RH, RT. The measurement was recorded in a ≈10s timeframe for computational processing and segmentation. The segmented peak in each frame is ≈1.33 Hz. c) Comparison of the established triboelectric series and triboelectric series of all users ranked according to the peak open‐circuit voltages of the materials in Figure 4b with corresponding material colors (orange represents PTFE, light blue represents silicone, red represents PET, dark green represents PE, light green represents cellulose, and dark blue represents PLA).

Here, we show the application of the body‐coupled triboelectric circuit for material differentiation captured with the contact‐separation mode in **Figure** **4**a. The triboelectric generation and collection processes were separated into two hands (collection at left hand and generation at the right hand) and thus mitigating the motion artefacts on triboelectric output as mentioned. Six types of materials were tested, which are silicone (Ecoflex), polylactic acid (PLA), cellulose (paper), polyethylene (PE), polyethylene terephthalate (PET), and polytetrafluoroethylene (PTFE) film, which were annotated from A to F, respectively. (As photographic images shown in Figure S7a, Supporting Information) Five participants were invited to the experiment and listed as User 1 to User 5. Under the test condition where the microfibers network parameter is N/d ≈60/1 mm, the average applied force of five users was set at ≈0.2 – 0.3 N at f ≈1.0 Hz and T ≈20 °C. The applied force was smaller than in Figure 2e due to the ease of controlling and regulating the tapping process for multiple materials. The triboelectric signal was captured by tapping the material in ≈10s and subsequently recorded and saved for computational segmentation. The distinct wave patterns and peak open‐circuit voltages of all users are shown in Figure 4b. As shown in Figure 1d, the body‐coupled triboelectric circuit consists of constantly shifting skin conditions, resulting in the dissimilarity of the signals captured by different users under the consistent settings. Taking the example of User 1, the highest open‐circuit voltages range from 0.043 V (PTFE) to 1.67 V (silicone), and the signal‐to‐noise (SNR) ratio increases from ≈2 (PTFE) to ≈8.3 (silicone) with the increase of signals captured.

The induced voltage magnitude reflects the difference in surface triboelectric charge density relative to human skin, and the signals are mostly aligned with the triboelectric series ranking in Figure 4c.^[^ ^58^, ^59^ ^]^ The triboelectric series of all users was compared, and the dissimilarity of the triboelectric series ranking of each user might be attributed to the difference in the human body and skin properties and conditions such as hydration level, skin elasticity, and roughness. Besides, the contact intimacy and material surface texture could also shift the ranking of materials in the established triboelectric series.^[^ ^59^, ^60^ ^]^ Notably, the voltage output of silicone film outperforms that of other materials owing to its soft and flexible elastomer nature. Softer PE foam showed generally higher triboelectric output than the rigid PTFE film despite its lower triboelectric negativity (Figure S7b, Supporting Information). Additional signal features like peak shape, negative‐to‐positive ratios, and sequence further distinguish material type patterns, revealing the capability of capturing material features. Finally, as shown in Figure S8 (Supporting Information), we also supplemented that the microfiber electrode can extract distinct triboelectric patterns when sliding over various surface micro‐textures, promising the application of capturing additional material surface information.

### 2.5 Machine Learning‐Assisted Tactile Augmentation

**Figure 5:**
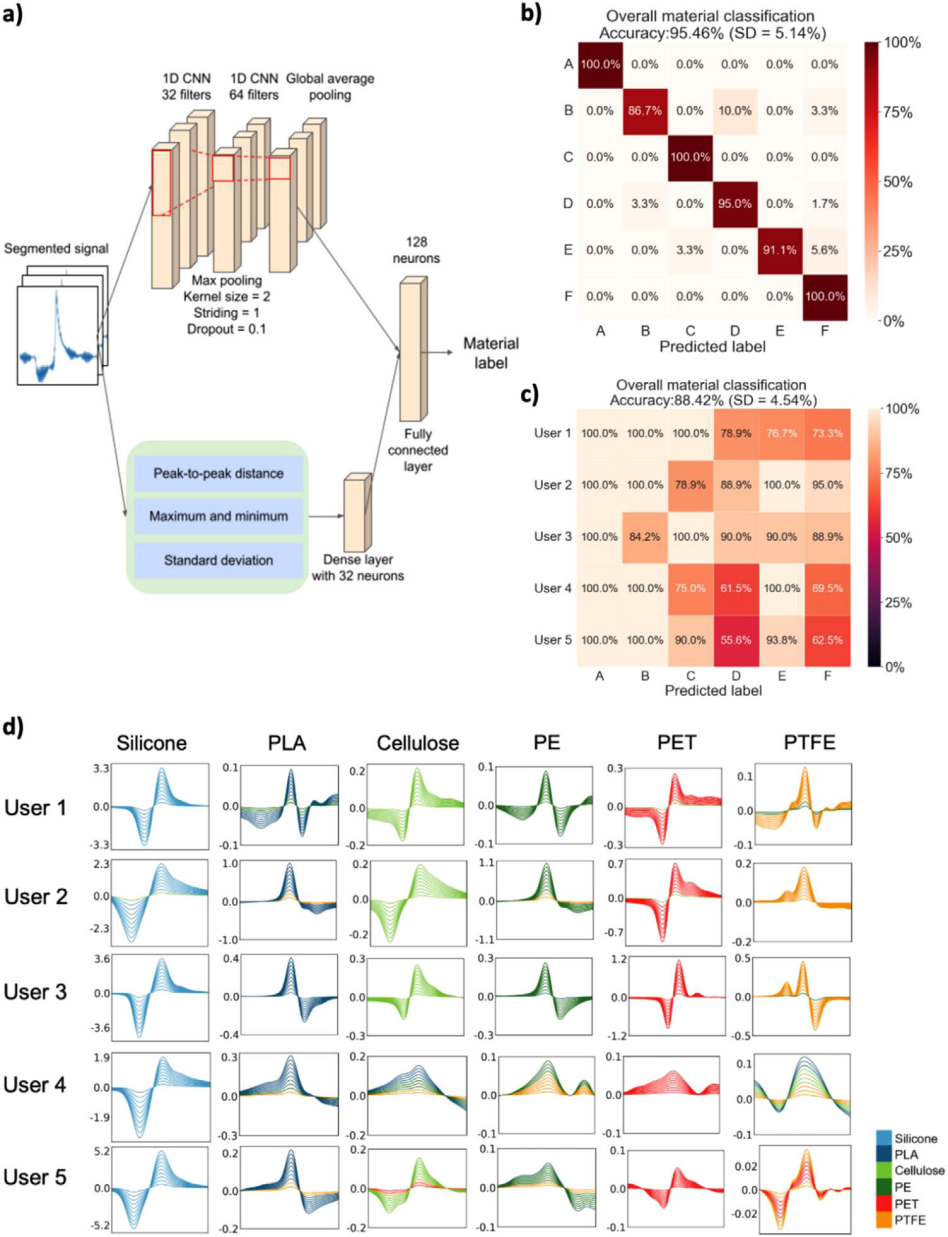
Machine‐learning assisted tactile augmentation. a) Flow diagram of wavelet decomposition and 1D CNN‐based machine learning model for materials and user recognition. b) Confusion matrices of machine learning results for classifying six materials with an overall accuracy of ≈95%, with a standard deviation of ≈5.1%. c) Confusion matrices of physical validation for material classification with an accuracy of ≈88% with a standard deviation of ≈4.5%. d) Signal boundary for each material of every user. The macro features of the average signal are scaled from 0.2 to 2.0 with a step size of 0.2. When the scaled signal reaches the boundary, another material that has the most similar feature, such as peak amplitude or number of peaks in the segmented signal, would be classified otherwise. The segmented peak in each frame is ≈1.33 Hz with *y*‐axis represented in voltage (V).

The microfiber electrodes enabled the collection of triboelectricity signals to describe the interactions between hand skins and external materials. To use this for tactile augmentation, rapid waveform recognition is required. However, the patterns are often embedded with noisy baselines and are unrecognizable to human eyes. Therefore, a machine learning model was integrated to explore its feasibility for high‐accuracy material recognition. To capture the spatial information, a feature‐ensembled 1D‐convolution neural network (CNN) was developed as shown in **Figure** **5**a. The architecture consists of a task‐specific input layer, followed by two convolution layers (with 32 and 64 neurons) with max‐pooling layers, a global average pooling layer, and fully connected final layers. For the task‐specific input layer, the inherent contact triboelectrification properties allow the signal macro‐features (e.g., amplitude, peak amplitudes ratio, envelope, etc.) to predict the six types of material. To enable the encoding of expert domain knowledge, customizable engineered input features such as descriptors of the pulses, were included.^[^ ^61^ ^]^ The features of the derivative of the waveform, the peak‐to‐peak interval, maximum peak value, minimum peak value, and the standard deviation were the most predictive. The details of data collection and pre‐processing were included in Note S2 (Supporting Information).

For the material classification among the six materials, the curated dataset consisting of six materials for at least an average of ≈20 samples per material type was collected from a single user with the model training process (Table S1, Supporting Information). For both datasets, the K‐fold test was used and 10% of the data was used as a test dataset while the rest was used for training the model within each fold. In the model training, the validation curves did not diverge from the learning curve after 50 epochs (Figure S9, Supporting Information). According to the machine learning results presented in Figure 5b, an accuracy of 95.46 % (SD = 5.14%) has been achieved for materials classification with six materials annotated from A to F, which corresponds to silicone, PLA, cellulose, PE, PET, and PTFE, respectively in the confusion matrix. The results of the individual confusion matrix are summarised in Figure S10 (Supporting Information). To validate the model accuracy in real‐world applications, the users were requested to tap the material in similar settings (F ≈0.2 – 0.3 N, f ≈1.0 Hz, Table S2, Supporting Information), with a validation accuracy up to 88.42 % displayed in Figure 5c. The relatively lower validation accuracy in this case can be ascribed to signal variance among users stemmed from dynamic skin conditions, differences in hand motion, and signal processing limitations. Ever‐changing skin properties affect charge generation, while inconsistent patting gestures alter signal patterns. Additionally, low signal output, such as PTFE for Users 4 and 5, is close to background noise, which complicates signal filtering and segmentation in the validation process, resulting in poor classification accuracy.

Figure 5d illustrates the signal boundary for each material of every user, further indicating how similar or distinct the signal patterns are. Materials with consistent, unique patterns (like Silicone and PTFE) were classified more accurately in panels 5b and 5c. In contrast, overlapping or variable signals (e.g., for Cellulose, PE, PET) led to more confusion, especially for Users 4 and 5—explaining their lower accuracy. This highlights the adverse impact of the human body induced variations of amplitude‐based features toward the model prediction. Apart from using amplitude for classification, signal shape and boundary were also taken into consideration when a segmented signal had an unknown amplitude range for the model, selecting the material that had the closest shape and thereby supporting the classification process.

Overall, the developed single‐channel CNN model achieved comparable accuracy with lower data and channel requirements, exceeding multi‐channel sensors and at least a hundred training samples per material, as reported in previous literature.^[^ ^25^, ^62^, ^63^ ^]^ The single‐channel strategy with low data requirements would be convenient for a better user experience. To streamline the process of real‐time material prediction, a pre‐trained model was deployed through a graphical user interface. The model revealed efficient and quick material classification of the material (silicone) by through hand contact‐separation, promising imperceptible tactile augmentation for the human‐machine interface (Figure S11 and Video S4, Supporting Information). However, variations in user‐specific interactions may still influence classification performance and microfiber applications. Further endeavors should focus on refining the signal pre‐processing steps and the machine learning model to improve sensitivity and resolution across diverse user profiles.

## 3 Conclusion and Outlook

This work reports an imperceptible microfiber electrode for human tactile augmentation. By utilizing the epidermal inherent electrical properties, the electrode enables efficient triboelectric signal extraction through a body‐coupled triboelectric circuit model. Tuning the microfiber electrode design could enable optimal output of the body's triboelectricity. Instead of utilizing or wearing external triboactive layers that cover the skin, we extract the intrinsic skin triboelectric signals, promising minimal obstruction to the skin sensation. Tests across various materials revealed distinct triboelectric patterns that could be further classified for specific material differentiation. A wavelet and feature engineering‐based 1D CNN model was employed to classify these signals. Ultimately, user‐specific body triboelectric responses were identified and categorized for tribo‐characteristics and material recognition.

Despite its advantages in leveraging the intrinsic triboelectricity of the human body, the current system may face challenges in extracting and classifying signals from materials with low voltage output. Future research should focus on enhancing the sensitivity of signal extraction and improving analytical models for more precise differentiation. Expanding material libraries and leveraging advanced machine learning techniques could bridge these gaps, enabling better performance in deploying human‐machine interfaces and paving the way for potential applications in medical assistance, augmented reality, and beyond.

## 4 Experimental Section

### Microfiber Solution Preparation

The whole process and orbital spinning setups were thoroughly reported in the previous literature.^[^ ^47^ ^]^ Briefly, the microfiber solution for orbital spinning was synthesized by mixing the 95%(v/v) PEDOT: PSS (poly (3,4‐ethylenedioxythiophene): polystyrene sulfonate) solution with polyethylene oxide (PEO) solution. The PEDOT: PSS dispersion was prepared by adding 5% (v/v) of ethylene glycol (Sigma–Aldrich) and 10 µL of dodecylbenzenesulfonic acid (Sigma‐Aldrich) to the 10 mL PEDOT: PSS solution to prevent aggregation. The solution was then sonicated in the water bath for 5 min. The PEO solution was prepared by dissolving 100 mg PEO (8m Da) and 25 mg of sodium hyaluronate into 5 mL of deionized water and stirring for ≈48h. Subsequently, the PEDOT:PSS and PEO solutions were mixed in a volume ratio of 2:1 and stirred for 12 h to be further orbitally tethered to the fingertip.

### Microfiber Electrode Printing Process

The orbital spinning platform for microfiber printing featured a cylindrical spinning zone adjustable from 0.5 to 15 cm in diameter and 30 cm in depth, powered by a servo motor (Parallax 6V continuous servo) operating at 45 – 65 rpm. The microfiber spinning platform, comprising the solution feeding system and rotating arm, was mounted on a translational stage. A 1 mL syringe connected to a 22‐gauge blunt‐end stainless‐steel needle (Adhesive Dispensing Ltd) was positioned above the rotating arm, allowing the pendant solution droplet to be engaged by the arm. Before the microfiber spinning process, two copper tap strips were overlapped to the surface of the fingernail with a conductive cable sandwiched inside, acting as signal transmission. For laboratory‐based experiments, the solution was fed into the syringe using a pressurized air supply (Elveflow OB1 microfluidic flow controller). The linear movement required for generating parallel fiber arrays with varying densities was regulated by a linear translational stage (Thorlabs MTS50‐Z8).

### Body Triboelectricity Measurement

The protocol for measuring the body triboelectric output was illustrated below: the left index fingertip was tethered with microfiber in a certain number of 60 and a width of 1 mm as the working electrode, and the left wrist was placed with a gel electrode (Covidien H124SG) as a reference electrode. In the case of excluding mechanical and electrical instability, the right hand and fingers were used to tap the materials with a frequency of ≈1.0 Hz and an applied force of ≈0.2 – 0.3 N. Moreover, the contact‐separation distance for the tapping process ranged from ≈15 – 20 cm. All materials and textures were tailored into a fixed size of 10 cm × 10 cm for standardized triboelectric output comparison. The body triboelectric voltage output was recorded by an oscilloscope (Keysight InfiniiVision DSOX2002A) with a positive probe connected to the fiber array and a negative probe to the reference electrode, respectively. As for all controlled parameter tests, the aforementioned tapping conditions were adopted, with temperature, force, and frequency parameters manipulated by placing a hotplate, scale, and commercial metronome underneath the materials respectively.

### Microfiber Electrode Stability and Repairability Study

The microfiber electrode repairing study was conducted by depositing ≈200 microfibers on the left index fingertip across a width of ≈4 mm (N/d ≈200/4 mm). Then, a cotton swab wet with deionized water was used to selectively wipe the fiber array on the skin‐nail interface back and forth until the microfiber was visibly undetectable, followed by printing new microfibers on the wiped area for 1 min to form a new sensing interface. The microfiber water resistance experiment was carried out by soaking the index finger in a beaker filled with deionized water, after which the finger was dried in the air for 30 min. The periodic friction test was implemented by pressing the finger on a plastic rotator at a mean normal force of 3 N. The rotator has a radius of 2 cm with a linear velocity of 4 cm s^−1^, and a red line marker was drawn on the rotator to count the friction cycle. The wearing stability was tested by spinning the fiber array of 60/1mm to the index fingertip, and the triboelectric output wearing time at 0, 6, and 12h were recorded correspondingly. All experiments utilized a silicone film (Ecoflex) for triboelectric output evaluation. The setup and test conditions of microfiber stability and repairability made no difference with the aforementioned tapping protocol.

### CNN Model and Algorithm

This Wavelet‐enhanced architecture of CNN maintains the complementary spatial and spectral resolutions for recognition. The architecture first involves encoding the 1D waveform into different down‐sampled representations (i.e., 64 representations given 64 kernels with different weight values, each with a dimension size of 1 x 2). The max pooling layer contributed to the filtering of only the maximum feature value propagating to the subsequent layer for down‐sampling and spatial abstraction. The output of the first convolutional pooling transformation feeds into a second set of convolution and pooling for further down‐sampling via secondary features extraction and spatial abstraction. To further improve accuracy by an extra 3‐5%, the wavelet‐based CNN auto‐extracted small features were then combined with engineered features encoded by a layer of 64 neurons, found to be predictive via backward feature selection method, for the final linear prediction layer prediction with various number of output nodes depending on the learning task. The learning algorithm involves iteratively minimizing the back‐propagation of the loss value based on the cross‐entropy loss function with x as the input feature values, the probability distribution of predicted values as p and ground truth as q, given the difference of distributions between the forward propagation values and the ground truth labels to ensure the convergence of the two distributions $y\;\approx \;\hat{y}=f\left(x|\theta_{fitted}\right)$ where y was the ground truth, $\hat{y}$ was the predicted value, *f*(*x*|θ_*fitted*_) was the model learnt based on the fitted weights θ_*fitted*_, and input feature *x*. After training the CNN model with the collected dataset, the CNN model was then applied to the typical input pattern (i.e., average signal), with a scaling factor from 0.2 to 2.0 with a step size of 0.2, to generate the signal boundary for each material for each user.

### Ethics Statement

Human participant experiments were performed with the approval of the Ethics Committee of the Department of Engineering at the University of Cambridge (7 July 2021, CUEDREC) and after obtaining informed consent from volunteers.

## Conflict of Interest

The authors declare no conflict of interest.

## Supporting information



Supporting Information

Supplemental Video 1

Supplemental Video 2

Supplemental Video 3

Supplemental Video 4

## Data Availability

The data that support the findings of this study are available from the corresponding author upon reasonable request.
