# Supplementary material for: Tactile Augmentation of Material Classification via Imperceptible On‐Skin Triboelectricity Collection
Source: Adv Sci (Weinh). 2025 Jul 2;12(31):e00217. doi: 10.1002/advs.202500217 (PMC12376505; doi:10.1002/advs.202500217)
Supplement: Supplementary file 1 — Supporting Information [file ADVS-12-e00217-s002.pdf]

ADVANCED SCIENCE
Open Access

Supporting Information

for *Adv. Sci.*, DOI 10.1002/advs.202500217

Tactile Augmentation of Material Classification via Imperceptible On-Skin Triboelectricity Collection

Junting Huang, Stanley Gong Sheng Ka, Haydn Cheong, Yaru Zhang, Daping Chu, Sohini Kar-Narayan, Wenyu Wang* and Yan Yan Shery Huang**

Supplementary Information

Tactile augmentation of material classification via imperceptible on-skin triboelectricity collection

Junting Huang[1,2]‡, Stanley Gong Sheng Ka[1,3]‡, Haydn Cheong[4], Yaru Zhang[5], Daping Chu[4], Sohini Kar-Narayan[3], Wenyu Wang[6]*, Yan Yan Shery Huang[1,3]**

[1] The Nanoscience Centre, University of Cambridge, 11 JJ Thomson Avenue, CB3 0FF, Cambridge, UK

[2] Department of Materials Science and Metallurgy, University of Cambridge; Cambridge, CB3 0FS, UK

[3] Department of Engineering, University of Cambridge, Trumpington Street, CB2 1PZ, Cambridge, UK

[4] Centre for Photonic Devices and Sensors, Department of Engineering, University of Cambridge, Cambridge, CB3 0FA, UK

[5] Department of Institute for Materials Discovery, University College London; London, WC1E 6BT, UK

[6] Thrust of Smart Manufacturing, Hong Kong University of Science and Technology (Guangzhou); Guangzhou, 511458, China

* Corresponding authors. Email: jh2448@cam.ac.uk; wenyuwang@hkust-gz.edu.cn; yysh2@cam.ac.uk

Key words: tactile augmentation, triboelectricity, fibers, material differentiation

This file includes:

Supplementary Note 1: Microfiber printing on fingertip as a multifunctional platform
Supplementary Note 2: Triboelectric data collection and pre-processing
Supplementary Table 1: Summary of samples of all users collected for computational processing
Supplementary Table 2: Summary of samples for physical validation by users
Fig. S1: Signal output of positioning electrodes in different skin locations
Fig. S2: Imperceptible microfiber electrode for unobstructed fingerprint unlocking of a smartphone
Fig. S3: External load resistance of fiber array in body-coupled triboelectric circuit
Fig. S4: Impedance comparison on different working electrodes placed on the fingertip, coupled with the same commercial gel electrode
Fig. S5: Photographic images of microfiber electrodes under different friction cycles
Fig. S6: Electromechanical characterization of microfiber via cyclic tapping
Fig. S7: Effect of material stiffness on captured triboelectricity signals
Fig. S8: Capturing surface texture triboelectric signals by hand sliding motion
Fig. S9: Learning curves of the machine learning model trained by the user's input
Fig. S10: Confusion matrices of five users for material classification in the model
Fig. S11: Photographic images of real-time material prediction through the computational model

Other Supplementary Materials for the manuscript include:

Movie S1 to S4

Supplementary Video 1: Imperceptible microfiber electrode for fingerprint unlocking.
Supplementary Video 2: Dry periodic friction test on the microfiber electrode
Supplementary Video 3: Water-soaking test on the microfiber electrode
Supplementary Video 4: Real-time material classification by contact-separation mode

Supplementary Note 1 Microfiber printing on fingertip as a multifunctional platform

The microfiber electrode enables multiple functionalities, including ECG (electrocardiogram) sensing, dual-sensing, mist sensing, and skin-gated OECT (organic electrochemical transistor) operation. For instance, the microfiber electrode enables "dual-sensing", meaning that upon touching another person, both people' ECG signals could be acquired by the microfiber arrays. These combined capabilities and multi-functionality further validate our decision to print the electrode on the fingertip, ensuring this microfiber array serves as a multifunctional platform for diverse applications. Moreover, the fingertip is primarily compatible with our fiber orbital spinning apparatus as it provides an easily accessible and non-intrusive site for electrode placement compared to other areas, such as the forearm, neck, and head, making it practical for wearable applications. The small surface area of the fingertip could also minimize the spinning time and material consumption while maintaining the same functionalities. Additionally, compared to areas like the forearm or wrist, the fingertip has fewer large muscle groups, reducing movement-related artefacts and improving signal quality.

Supplementary Note 2. Triboelectric data collection and pre-processing

The curated dataset consists of a total of 647 samples (~65 samples for testing) for all materials. For each material, the collected dataset has a total of less than 150 samples. For testing, 10% of the data was held-out following 3-folds scheme while keeping the rest for training the model and tuning the hyperparameters. The ML workflow consists of four fundamental stages, which are described as (1) data collection and preprocessing, (2) features establishment and baseline model, (3) model selection, training, and validation, and (4) deployment for real-time usage. For the data collection and preprocessing stage, a Python program was created to directly stream the sensor data stream from the oscilloscope to the connected PC with initial low-pass filtering (cutoff frequency at 20 Hz) and threshold-based segmentation for touch event detection, followed by waveform visualization for user confirmation before loading into the model training dataset. The target class labels were encoded into one-hot encoding using the Scikit-learn package. For visualizations, Matplotlib, and Seaborn packages were used for the visualizing of pulses, learning curves, and confusion matrices. TensorFlow Keras 2.6.0 was used for the deep learning model. Initially, the commonly reported descriptive features were used, and then backward elimination was used for feature selection. The descriptive features found to be contributing to the overall increase in prediction accuracy include the positive/negative peak amplitudes, peak-to-peak interval, peak-to-peak ratio, and derivative.

Supplementary Table 1. Summary of samples of all users collected for computational processing.

User	Silicone	PLA	Cellulose	PE	PET	PTFE
**1**	19	19	21	20	20	20
**2**	18	28	27	20	22	17
**3**	18	37	18	20	18	20
**4**	16	18	13	14	21	42
**5**	18	21	29	13	29	31
**Total**	89	123	108	87	110	130
**Average per material**	14.8	20.5	18.0	14.5	18.3	21.7

Supplementary Table 2. Summary of samples for physical validation by users.

User	Silicone	PLA	Cellulose	PE	PET	PTFE
**1**	10	7	8	19	30	30
**2**	9	9	19	9	10	20
**3**	10	19	10	10	10	9
**4**	8	14	16	16	16	82
**5**	11	10	10	18	16	80
**Total**	38	59	63	63	82	221

In Fig.5c, as mentioned in Section 2.4, triboelectrically negative yet stiffer material, such as PTFE, in general, is more difficult to classify even though more validation tests were conducted compared to other materials. The recorded signals often consist of low peak amplitudes (< 0.01V) and could not be segmented by the algorithm, resulting in much lower accuracy than other materials.

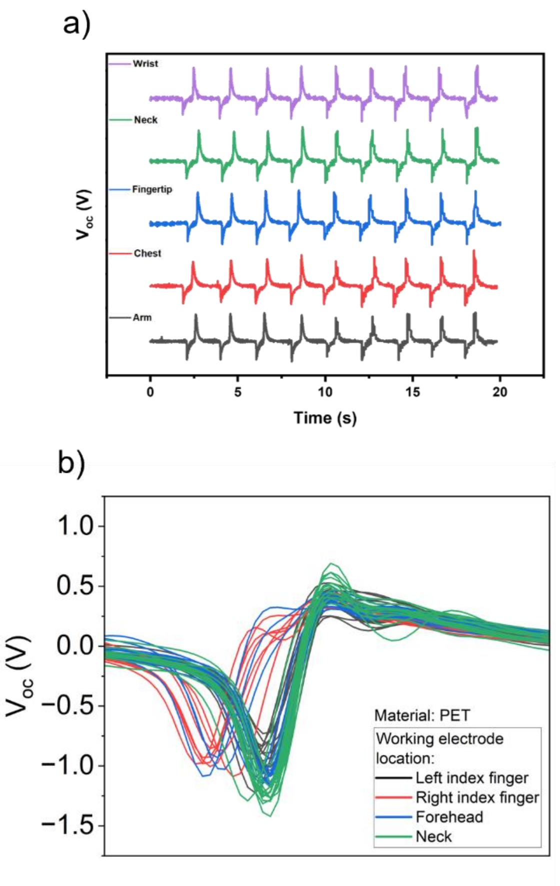

Figure S1. Signal output of positioning electrodes in different skin locations. a) By changing the skin locations of reference gel electrodes, consistent triboelectricity signal features (positive and negative peaks) with similar amplitudes could be captured. b) The triboelectricity test is conducted using PET while attaching the working gel electrodes in different skin locations. The signals are then segmented and compared, showing similar peak features and amplitudes (~-1.25V to ~0.5V). Overall, the location of fiber electrode or reference electrode does not compromise comprehensive capture of the triboelectricity signal. This could be partially explained by the special body circuitry, non-linearity and similarity of the human body impedance[1], as well as the close fiber contact impedance on different body part.

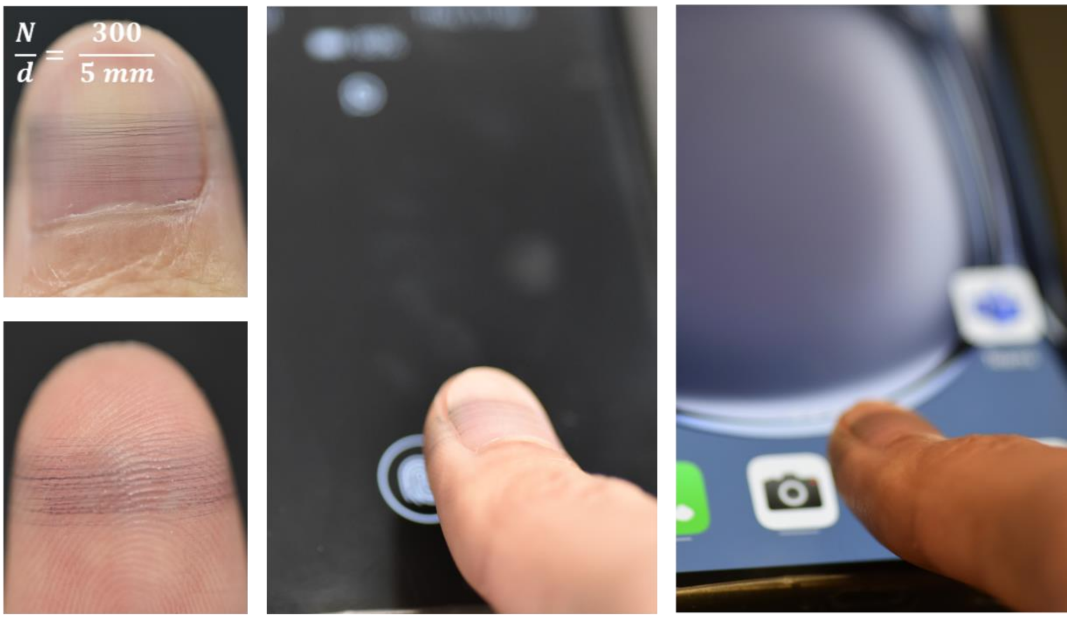

Figure S2. Imperceptible microfiber electrode for unobstructed fingerprint unlocking of a smartphone.

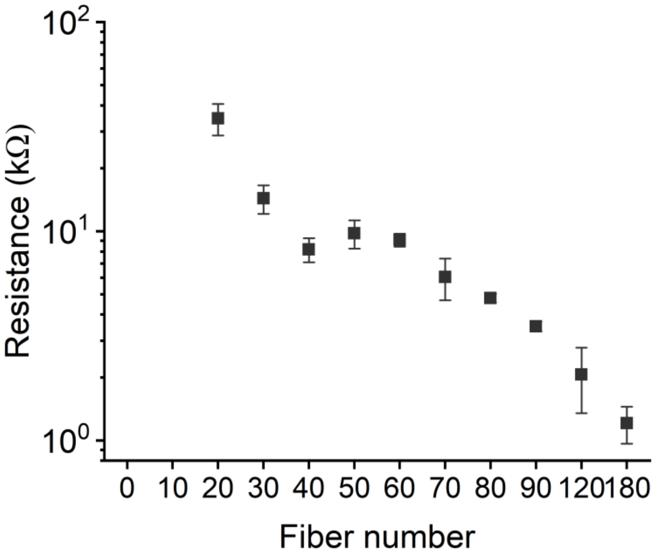

Figure S3. The external load resistance of fiber array in body-coupled triboelectric circuit. The fiber resistance is measured from N/d ~ 20/1mm to 180/1mm. Increasing the fiber density would decrease the overall external load resistance. According to triboelectric efficiency theory[2], voltage amplitude is positively correlated with external load resistance, resulting in a diminished voltage output at high fiber number density.

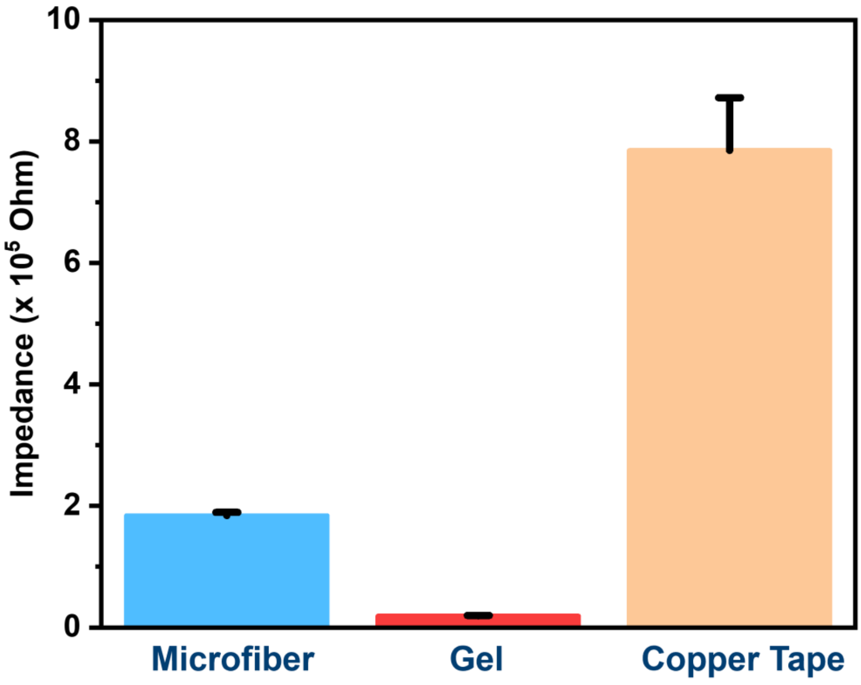

Figure S4. Impedance comparison on different working electrodes placed on the fingertip, coupled with the same commercial gel electrode put on the wrist for reference. The impedance was measured at 1k Hz. Microfiber number density: 60/1 mm. The copper tape electrode shows the largest impedance with lower voltage output and an unstable background. The microfiber electrode shows a lower impedance for increased voltage output. Gel electrodes were used, where one was put on the wrist while the other was put on the index fingertip, to measure the skin impedance for reference. Impedance results were measured at least 5 times for each working electrode with error bars (standard deviation).

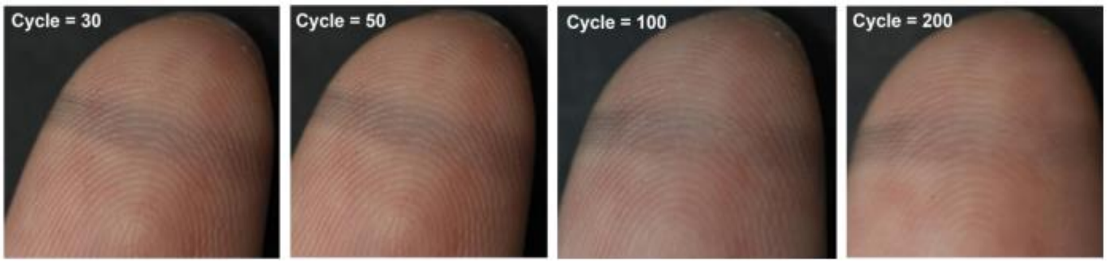

Figure S5. Photographic images of the microfiber electrode under different friction cycles.

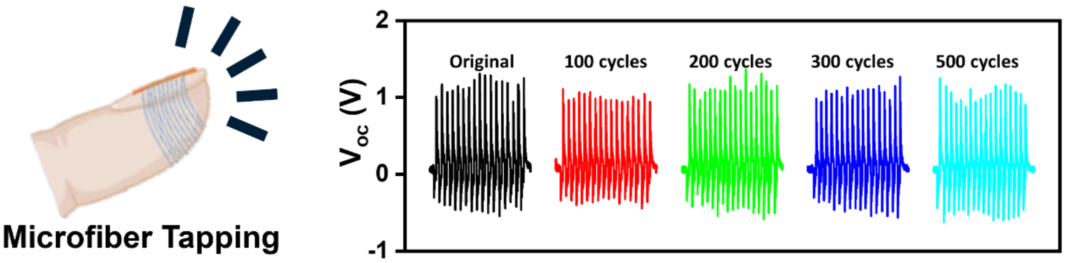

Figure S6. Electromechanical characterization of microfiber via cyclic tapping. The open-circuit voltage output on different cycles of a fingertip wrapped with microfibers (fiber number density = 60/1 mm) tapping on the silicone film.

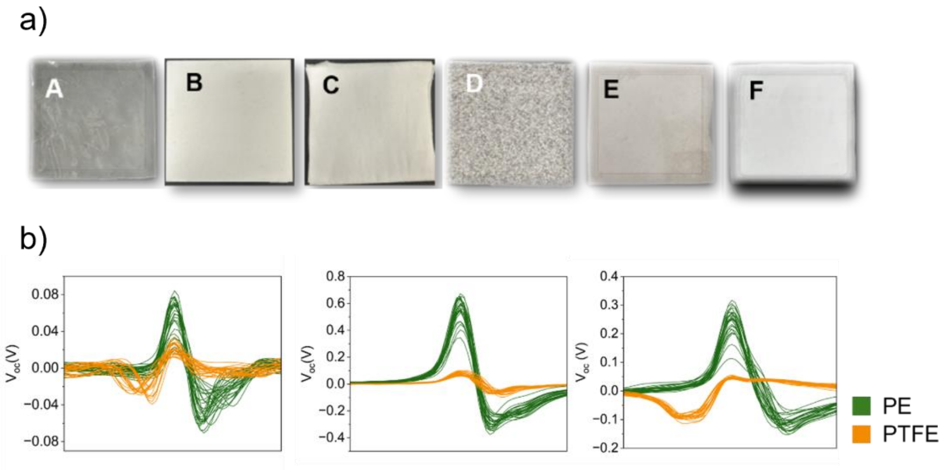

Figure S7. Effect of material stiffness on captured triboelectricity signals. a) Photographic images of all materials (A: silicone, B: PLA, C: cellulose, D: PE, E: PET, and F: PTFE). b) Comparison of PE and PET triboelectricity signals from three newly invited users. All PE signals are larger than PTFE signals, showing consistent triboelectric behaviour of the materials tested.

In our personalized triboelectric series, PE is more triboelectric negative than PTFE. This is because PTFE is a solid film with low elasticity, whereas PE is structured as a foam. The porous nature of PE foam significantly increases the effective contact area with human skin, resulting in higher triboelectric voltage output. Conversely, PTFE, being a smooth and rigid plastic film, exhibits lower contact intimacy with the human hand, leading to reduced voltage output. Ecoflex silicone film demonstrated the highest voltage output since it is highly elastic and slightly adhesive, maximizing contact intimacy with human skin and enhancing charge generation. Moreover, the dielectric properties and exact composition of the material surface could vary from the pure material as labeled, shifting it from the established triboelectric series.

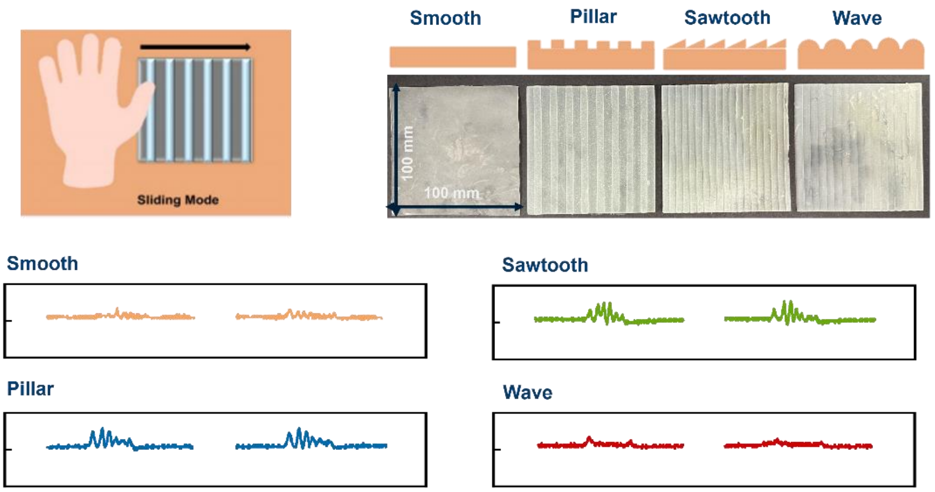

Figure S8. Capturing surface texture triboelectric signals by hand sliding motion. Four silicon films (10cm × 10 cm) are fabricated with different micro-textures: smooth, pillar, sawtooth, and wave. The sliding test was conducted at a sliding rate of ~ 5cm/s and a normal force of ~ 1 N. The x-scale is from 0 - 10 s, and the y-scale is from -1 - 1 V for each frame.

However, the signal quality from the body-coupled circuit was relatively low in the surface micro-texture differentiation, making segmentation and classification challenging. Variability such as sliding speed, force, and angle, among users also introduced signal inconsistencies, making the sliding test harder to standardize than the contact-separation mode.

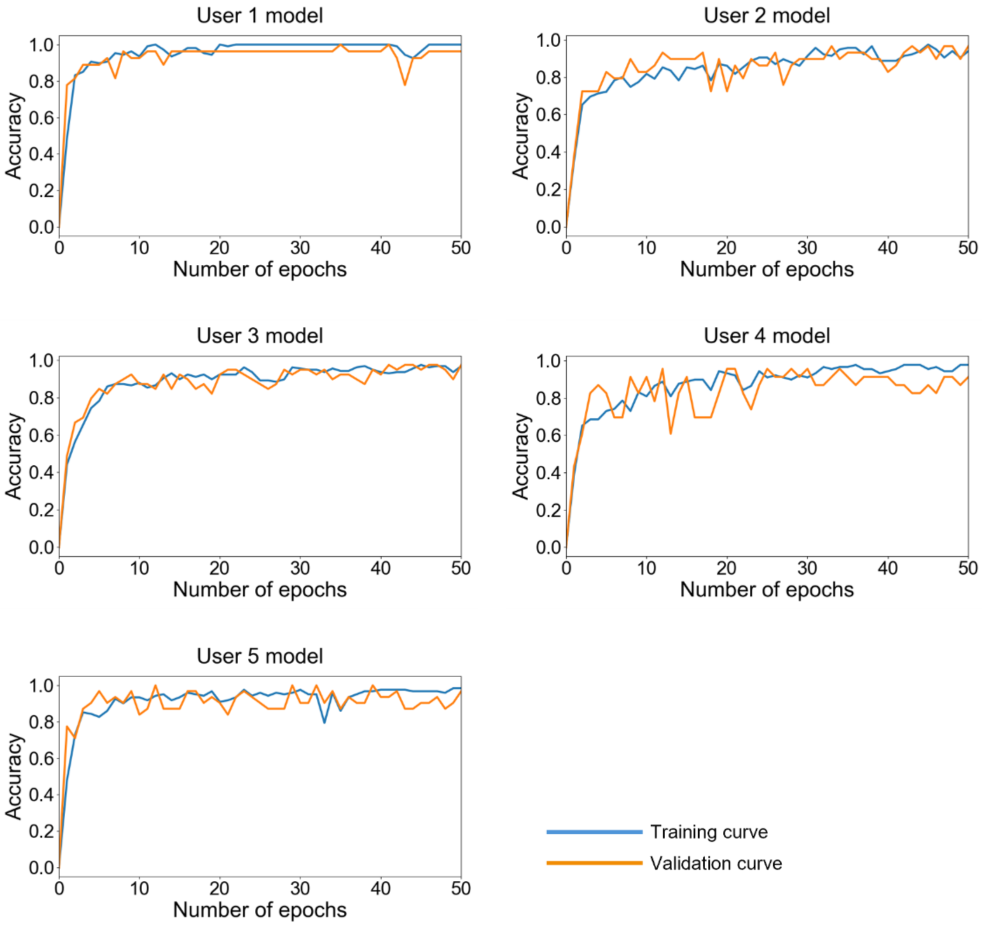

Figure S9. Learning curves of the machine learning model trained by the user's input. For all users, both the training curve (blue line) and validation curve (orange line) showed improvement and convergence of accuracy over the 50 epochs (i.e., the number of scanning through the entire dataset for backpropagation of network weights). The validation curve did not diverge from the training curve and indicated that overfitting was not presented.

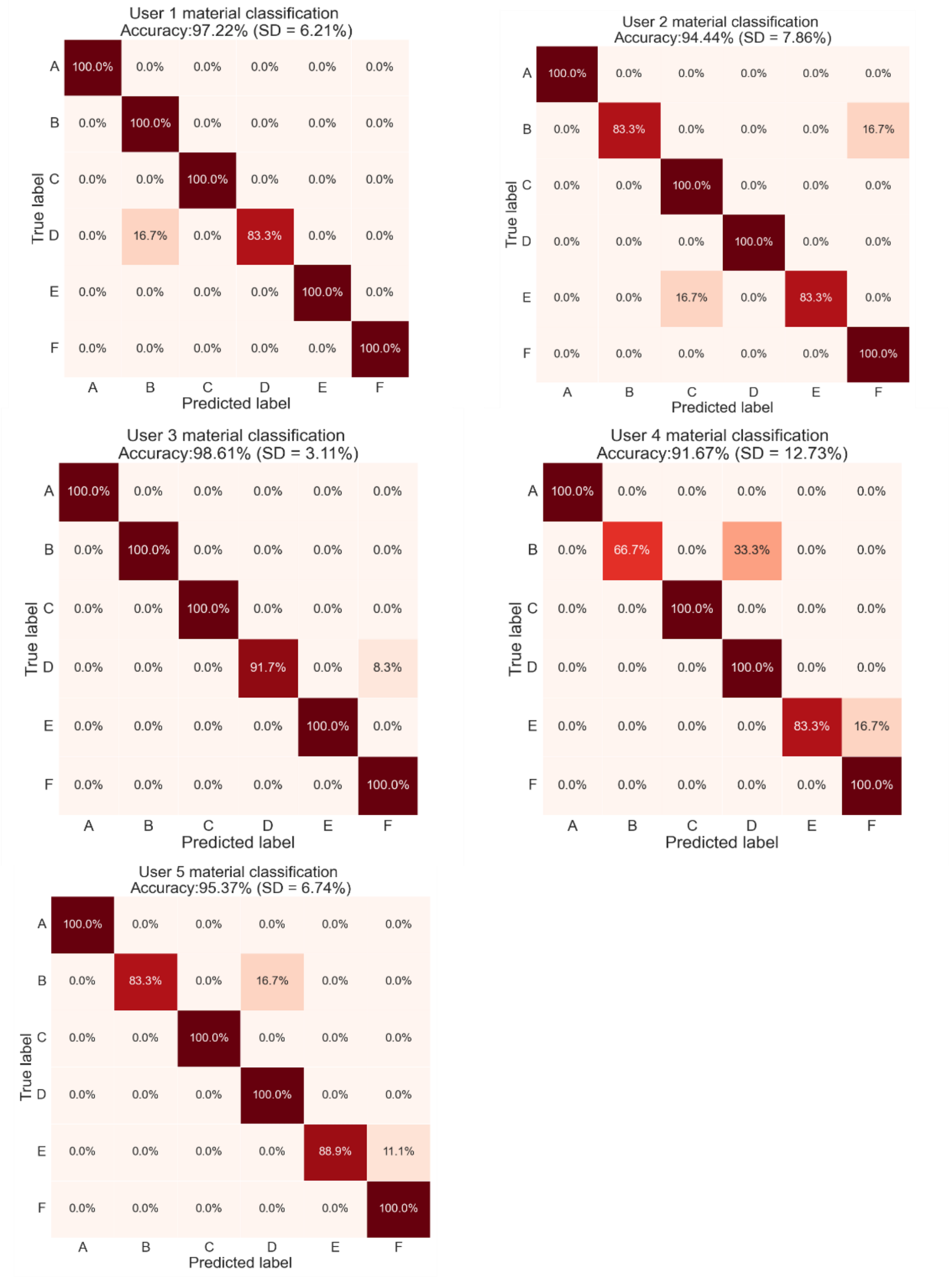

Figure S10. Confusion matrices of five users for material classification in the model. The matrices show that the machine learning model is capable of differentiating the materials tested with an accuracy of at least 90%.

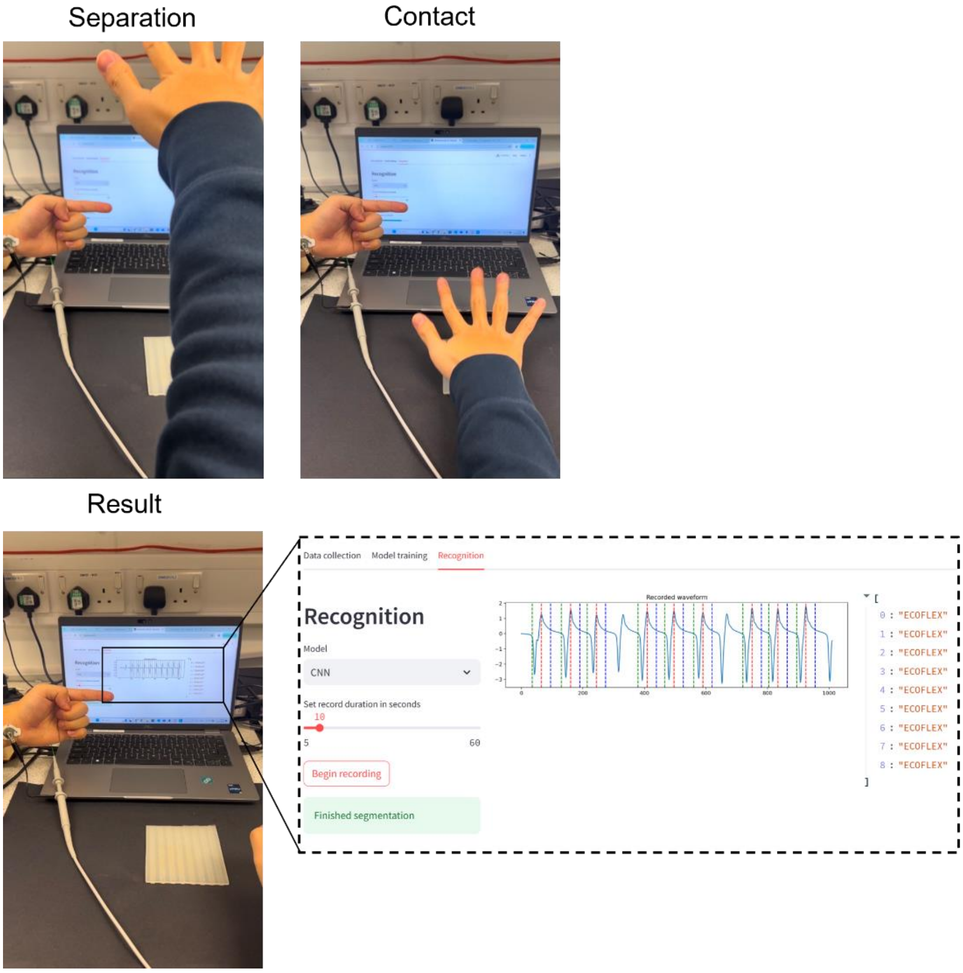

Figure S11. Photographic images of real-time material prediction through the computational model. The fibre electrode and the reference electrode were both on the left hand for triboelectricity collection while the right hand was for triboelectricity generation. After tapping the material by contact-separation mode for ~ 10s, the model prediction displays the correct material.

Reference:

[1] D. J. Bora, R. Dasgupta, *IET Syst. Biol.* **2020**, 14, 230-240.

[2] H. Wu, C. Shan, S. Fu, K. Li, J. Wang, S. Xu, G. Li, Q. Zhao, H. Guo, C. Hu, *Nat. Commun.* **2024**, 15, 6558.
